# Group Size Dependent Selection for Cooperation Versus Freeloading in Collective Chemical Defence

**DOI:** 10.1111/ele.70378

**Published:** 2026-04-16

**Authors:** Sophie Van Meyel, Raphael Ritter, Heikki Helanterä, Carita Lindstedt

**Affiliations:** ^1^ Department of Forest Sciences University of Helsinki Helsinki Finland; ^2^ Ecology and Genetics Research Unit University of Oulu Oulu Finland

**Keywords:** group living, hymenoptera, predation, public goods, sociality

## Abstract

Cooperation in public goods is expected to evolve more rapidly in smaller groups than in larger groups because individuals receive a larger share of the benefits, reducing the benefits of freeloading. However, experimental evidence for this hypothesis remains limited to microorganisms, restricting our understanding of the evolution of cooperative traits. Here, we show that in the collectively defending larvae of *Neodiprion sertifer* , survival against predation is higher in cooperative groups, with benefits of cooperation more pronounced in small groups (5 larvae) than in large groups (20 larvae). Individuals also participate less in collective defence in larger groups, not because of higher life‐history costs resulting from increased resource competition but because they adjust their contribution according to group size. These results provide novel empirical evidence that selection for cooperation in collective goods is group size‐dependent, promoting cooperation in smaller groups, whereas the relative fitness of freeloaders is higher in larger groups.

## Graphical abstract

Theory predicts that cooperation in public goods should be favoured in smaller groups because individuals gain a larger share of the benefits, but empirical evidence remains scarce outside microbial systems. Using collectively defending larvae of the pine sawfly *Neodiprion sertifer* , we show that survival benefits of cooperation are stronger in small than in large groups, and that individuals reduce their defensive investment in larger groups by adjusting their contribution to group size. These results provide direct empirical evidence that selection on cooperative investment is group size‐dependent.

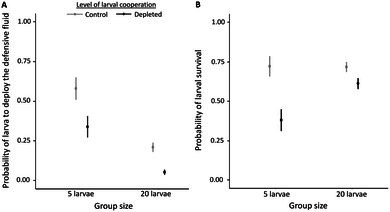

## 1 Introduction

Evolution of group living and cooperation within a group has played a pivotal role in the evolution of life. For example, in groups individuals can effectively acquire and share resources that are costly to acquire, such as food (Meurville and LeBoeuf 2021), enzymes (West et al. 2006) or defensive compounds against predators and pathogens (Cremer et al. 2018; Smith and Schuster 2019) known as *public goods*. Cooperation in public goods can evolve when sharing resources generates higher net benefits for an individual compared to what it can achieve alone (West et al. 2007). For example, in *Pseudomonas aeruginosa* bacteria, defensive bacteriocin compounds against competing microorganisms are effective only when their concentration surpasses a critical threshold, requiring cooperation among multiple bacterial cells (Juhas et al. 2005). However, contributions to public goods are often facultative, allowing individuals the opportunity to adjust their investment to the prevailing ecological context (Lindstedt et al. 2018; Smith and Schuster 2019). Since the benefits of public goods are shared within the local group, individuals that do not pay the costs of cooperation (freeloaders) can still benefit from investment by other group members. This makes public goods systems often vulnerable to freeloading which can corrupt cooperation (Ghoul et al. 2013).

One area of interest in theoretical research has been the effect of group size on the evolution of cooperation (Hauert and Doebeli 2004; Lehmann and Keller 2006). Cooperation is expected to evolve more readily in smaller groups than in larger ones, particularly when the benefits of cooperation are shared among all group members (Archetti 2009). In smaller groups, each individual receives a larger share of the collective benefits (1/n) and, as the group size increases, an individual's contribution to public goods has a smaller impact on the overall outcome, while costs to produce the cooperative act remain the same. As a result, in larger groups a cooperator receives a smaller share of the total benefits, reducing its pay‐off from the cost of contributing (Ross‐Gillespie et al. 2009). Furthermore, if individuals are also in higher density in larger groups, closer interactions between freeloaders and cooperators may be facilitated, increasing the prevalence of exploitation (Ross‐Gillespie et al. 2009). Cooperativeness can also decrease in larger groups if contribution to the public goods becomes more costly at higher densities, for example, due to the limitations in the availability of energy or resources that individuals can allocate on cooperative acts (Krausz 2013).

Currently, most of the empirical evidence for the ‘Group size hypothesis’ comes from microbial systems, where both population density (e.g., through dilution) and the relative frequencies of genetic strains of freeloaders and cooperators can be experimentally manipulated to assess the fitness of these two strategies across generations (Ross‐Gillespie et al. 2007, 2009). While density‐ and frequency‐dependent effects have been clearly demonstrated in these studies, they rely on genetically fixed cooperator and cheaters strains. Although bacteria can plastically adjust their cooperative investment (Kümmerli et al. 2009), group‐size effects on such plastic responses have not been directly tested. In addition, empirical evidence for the role of group size in shaping facultative cooperative acts outside microbial systems remains lacking. This limits our understanding of the evolution and selection of facultative cooperative traits, particularly when individuals can plastically adjust their level of contribution. This has been partly due to the challenges associated with experimentally manipulating both the group size that individuals occur in and their level of contribution to cooperation. This issue is particularly evident in vertebrate and insect study systems, where facultative cooperation is more prevalent.

**FIGURE 1:**
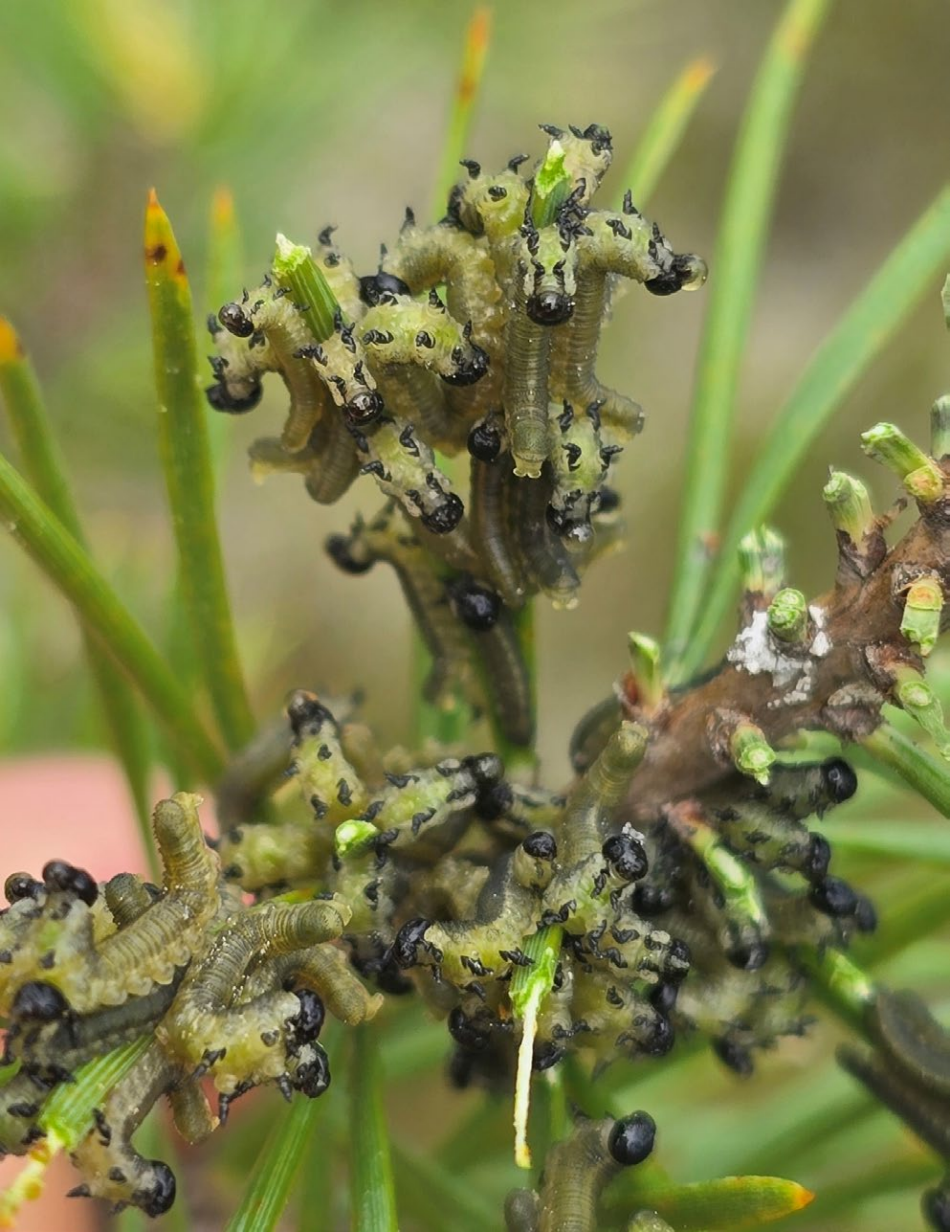
Group of defending larvae on a pine branch displaying their defensive behaviour in the wild. Some larvae display only a U‐shaped defensive posture, while others combine it with the deployment of defensive fluid. Photo credit Raphael Ritter.

Here we solve this problem by using the gregarious pine sawflies (Hymenoptera: Diprionidae) as a study system. This system allows manipulation of both the group size and the level of cooperation and the quantification of the costs and benefits of public goods cooperation for individuals under different environmental contexts (Lindstedt et al. 2018, 2025). The larvae of this species live in dense groups ranging from 5 to over 100 individuals, where they defend collectively chemically against predators (Figure 1) (Codella and Raffa 1995; Lindstedt et al. 2006). When threatened, the larvae display a defensive posture synchronously as a first line of defence, where they raise their head and posterior abdomen (U‐posture). The U‐posture makes larvae alert and is a prerequisite for them to move on for the second and more costly line of defence to deploy a terpene and resin‐rich fluid from their mouths (Codella and Raffa 1996; Lindstedt et al. 2025). These behaviours are shown in Videos S1 and S2. Defensive fluid from multiple pine sawfly larvae forms a sticky barrier, hindering predators from capturing larvae without becoming coated in the fluid (Codella and Raffa 1996; Eisner et al. 1974).

In general, these kinds of responsive chemical defences are considered to be public goods providing both individual benefits by increasing the survival of defending individual (Ritter et al. 2025; Skelhorn and Rowe 2006) as well as group‐level benefits by deterring predator more effectively (Daly et al. 2012; Speed et al. 2012). Prey individuals with responsive defences also share the costs of educating predators to avoid prey with similar appearances in future encounters (Jones et al. 2016; Speed et al. 2012). Additionally, chemically defended prey will benefit from being aggregated, as predators are less likely to attack prey individuals in groups where some or all group members are chemically defended (Jones et al. 2016; Mappes et al. 1999). Accordingly, pine sawfly larvae are more likely to survive in groups with higher proportion of defending larvae (Eisner et al. 1974; Lindstedt et al. 2006). In the wild, larvae occur primarily in kin‐groups. Therefore, deployment of defensive fluid provides also indirect benefits via increased survival of relatives (Ritter et al. 2025). However, deploying and losing the fluid repetitively has shown to be costly for pine sawfly larvae, decreasing, for example, their growth as well as their future defence ability (Lindstedt et al. 2018). The deployment of defensive fluid is also a facultative trait and larvae that deploy the fluid but do not lose it can reabsorb and reuse it. Furthermore, some larvae consistently refrain from deploying the fluid (so called ‘nil‐individuals’) and benefit by growing faster; these individuals are suggested to be freeloaders (Lindstedt et al. 2018). Finally, contribution to the defence is female‐biased as female larvae are more likely to deploy the fluid than males and produce higher volumes of it (Lindstedt et al. 2018; Ritter et al. 2025).

We used the above characteristics of *N. sertifer* to study experimentally (1) how group size and levels of cooperation, separately and in combination, influence survival against predators in a natural setting, (2) how contributions to collective chemical defence depend on group size and (3) how group size affects the performance and life‐history traits of individuals within groups. We predict that the survival benefits of cooperation should be more pronounced in smaller than in larger groups and, conversely, that the costs of freeloading should be lower in larger groups than in smaller groups. This is because defence may be less frequently required in larger groups because predation risk per se may decrease as group size increases (dilution effect) (Sillén‐Tullberg 1990). But see (Koskenpato et al. 2026; Lindstedt et al. 2006, 2011). Furthermore if individuals decrease their contribution toward costly cooperation in larger groups, the proportion of individuals exhibiting defence should be lower in larger than in smaller groups. Finally, if the costs of contributing to the chemical defence are higher in larger groups due to, for example, smaller amount of resources or energy per capita, individuals should show decreased growth in larger groups (Daly et al. 2012).

## 2 Methods

### 2.1 Effect of Group Size and Levels of Cooperation on Larval Survival Against Predators

In June 2023, 19 larval groups of approximately second instar *Neodiprion sertifer* were collected from Puumala, eastern Finland (61.56082, 28.01005). Larvae were fed *ad libitum* and maintained in standardised laboratory conditions (+20°C). When the larvae were between the 3rd and 4th instars, the 19 initial groups were randomly divided and split into two of the treatment groups, with the exception of one large larval group that had enough larvae for all four treatment groups. Thus, altogether, both depleted and non‐depleted groups of 5 and 20 larvae were created, resulting in 10 larval groups per each treatment, respectively. In depletion treatments, larvae were pressed with a cotton swab on the dorsal side. When they deployed their defensive fluid, it was removed with the swab. This was repeated twice on the following day. After the depletions, larvae were placed back into their experimental box and left overnight to re‐aggregate. On the following day, we tested the group defence by randomly choosing one larva per group and gently poking it once on the dorsal side with tweezers. We then scored the number of larvae exhibiting the U‐posture, and/or deploying defensive fluid.

To assess larval survival against predation, groups of larvae on their original pine twigs were attached to a 40 cm long pine branch with two smaller twigs and were installed 2–5 m from an ant nest trail into the field. Altogether, we used 10 different wood ant nests (*Formica s.str*.). All four treatments were tested at each ant's nest to take into account potential variation in activity or aggressiveness among ant nests. The survival of larvae was recorded by counting the number of larvae within each group at the beginning of the experiment and again after 7 h.

#### 2.1.1 Statistical Analyses for the Predation Experiment

To confirm the success of the depletion treatment, we first tested whether the probability of displaying the U‐posture (with or without fluid) and deploying the defensive fluid differed between group sizes and depletion treatments. The U‐posture (1 = yes, 0 = no) was analysed with a Bayesian GLMM with a Bernoulli distribution appropriate for binary (0/1 per larva) data and clog‐log link function (see Methods S2). The U‐posture was the response variable and group size (5 larvae, 20 larvae), depletion treatment (control, depleted) and their interaction were used as fixed effects. The larval group ID was included as a random effect to consider potential influences of the common environment effects of larval group. Details on priors and Hamiltonian Monte Carlo (HMC) settings are provided in Methods S2.

To analyse the probability of larval survival against ant predation in different treatment groups, we used a GLMM with binomial error distribution and a cloglog link function. Larval survival (1 = alive or 0 = dead) was used as the response variable and the depletion treatment and group size and their interactions were fitted as explanatory variables. Nest ID and larval group ID were included as random factors to consider potential variation in survival due to differences (e.g., activity and hunger level) among ant trails or ant nests.

### 2.2 Effect of Group Size on the Life‐History Traits and Contribution to Collective Chemical Defence

#### 2.2.1 *Neodiprion sertifer* Insect Cultures

The experiment was conducted in Jyväskylä, Finland. All *N. sertifer* families descend from an outbred F1‐generation of laboratory population established in 2016 and originated from 86 wild larval groups (58 groups from Puumala and 28 groups from Pieksämäki, Central Finland) (see Methods S1 for full rearing history). In May 2017, we collected pine branches with eggs which had been laid by females previously paired on mesh‐bagged branches (see Methods S1) and maintained the branches in a constant temperature with water (+20°C) until eggs hatched. Hatched larvae were reared under laboratory conditions with constant temperature (+20°C ± 2°C) and both natural light coming from the windows (nights are very short in Finland during May and June) plus artificial light at all times, reflecting the normal central Finnish summer light conditions. Larvae were fed *ad libitum* with fresh pine branches twice per week.

#### 2.2.2 Experimental Design

Families with the minimum of 40 individuals were chosen for the experiment to be able to follow a full‐sibling design. At the age of 10 days, 36 larvae per family were divided into three different group sizes: two groups of 3 larvae, one group of 10 larvae and one group of 20 larvae. We had two replicates per family of the 3 larvae—treatment to account for the unbalanced number of individuals across group size treatments. Larvae were kept in transparent plastic containers with fabric on top for ventilation in a condition described above. Altogether we had 16 families in the experiment from which 7 were F1 from Puumala and 9 were F1 from Pieksämäki. This resulted in *N* = 27, *N* = 15 and *N* = 13 for larval groups in treatments of 3 larvae, 10 larvae and 20 larvae, respectively. All the treatment groups included approximately similar amounts of females and males as group size treatments did not differ in terms of sex ratio (LR‐χ^2^ = 0.39, df = 2, *p* = 0.825).

#### 2.2.3 Contribution to Collective Defence Under Different Group Size Treatments

The group defence behaviour of larvae was measured on the third day of the experiment similarly to above. We recorded the number of larvae that adopted the U‐posture and the number of these that deployed the defensive fluid. We also measured the individual level contribution to the collective defence from the last larval instar at 16 days of age. For this part of the experiment, we used larvae from 14 families in total (*N* = 7 from Puumala and *N* = 7 from Pieksämäki). Each larva was individually gently pressed on the dorsal and ventral side with a capillary tube. We recorded whether or not the larva produced a defensive droplet. To measure the volume of any fluid produced, it was sucked into a 5 μL capillary tube (Microcaps, Drummond Scientific Co., Broomall, Penn.) the length of the liquid measured (using a digital calliper to the closest 0.1 mm) and converted to a volume.

#### 2.2.4 Effect of Group Size Treatment on Life‐History Traits

We recorded the development time of larvae to reach pupation (in days) and pupa weight (3–4 days after their pupation). To measure the reproductive output of females, females from different treatments were mated with males originating from the same treatment. Altogether, we had 31 pairs from groups of 3 larvae, 33 from groups of 10 larvae and 37 from groups of 20 larvae (for further information see Methods S1). Mated pairs from different treatments were put on the same tree on the same day (one pair per each treatment per one host tree) to control for possible variation due to host plant quality. Similarly, mated pairs were taken outdoors to lay eggs in a similar weather condition (sunny and dry days, +18°C to 25°C).

#### 2.2.5 Statistical Analyses of the Chemical Defence and Life‐History Traits

We used generalized mixed models to estimate how group size affects larval contribution to group defence. Display of the U‐posture (1 = yes, 0 = no) and deployment of the defensive fluid (1 = yes, 0 = no) were treated as dichotomous variables modelled as binomial response variables with a cloglog‐function. We also analysed the group level defence using the absolute number of larvae per group that performed the U‐posture and the number that deployed defensive fluid (both integer‐valued count data). For both traits, we used GLMM with Poisson error distribution and log‐link function. Each behaviour was modelled separately as the response variable, with group size as the only fixed effect. While probability models describe an individual‐level contribution in group defence, the absolute number models reflect the total group‐level mobilisation and allow us to distinguish between individual decision‐making and potential collective thresholds in group defence. In all the models, the population ID, family ID and group ID were included as random factors, to take into account any similarity due to common inheritances and environments.

To analyse how group size, sex and their interaction affects an individual's fluid deployment and its volume (i.e., when individuals were directly attacked and defending on themselves), we used a Bayesian approach using the brms package (Stan) (see above). We fitted a Bayesian GLMM with a Bernoulli distribution and cloglog link function. Deployment of the defensive fluid (1 = yes, 0 = no) was the response variable and group size (3 larvae, 10 larvae and 20 larvae), sex (female and male) and their interaction were entered as fixed effects. Population ID, family ID and group ID were included as random factors (details on priors and HMC settings are provided in S2). For the volume of fluid, the same fixed and random effect's structure and priors were used as above. We fitted a Bayesian GLMM with a gamma error distribution and a log link function since the data were positively skewed. Because the gamma distribution does not allow zeros, and measurement precision was 0.01, we used ‘measured volume + 0.01’ as the response variable (Lindstedt et al. 2018).

Due to sexual dimorphism, life‐history traits were also analysed separately for females and males (Lindstedt et al. 2018). We fitted three generalized linear mixed‐effects models (LMERs) with development time, pupa mass and egg production (the latter only for females) as response variables. To test the effect of the defensive behaviour (1 = yes or 0 = no) on an individual's life history traits, we included the defensive behaviour as a fixed factor together with the group size and their interactions.

All statistical analyses were performed in R v4.4.1 (http://www.r‐project.org/). When an interaction was not significant, we removed it and refitted a simplified model without the interaction term. For models where an interaction was significant, we performed pairwise comparisons between treatments using estimated marginal means (emmeans) and applied Tukey corrections for multiple comparisons (Dunnett 1955). For more detailed information, see Methods S2.

## 3 Results

### 3.1 Benefits of Cooperation Against Predators Are More Pronounced in Small Groups Than in Large Groups

We first examined how defensive behaviours (U‐posture and deployment of fluid) varied with group size and depletion treatment. Larvae in larger groups were less likely to display the defensive U‐posture than those in smaller groups (LR‐χ^2^ = 21.24, df = 1, *p* < 0.0001). The depletion treatment had no effect on the probability of displaying the U‐posture (LR‐χ^2^ = 1.58, df = 1, *p* = 0.21) and there was no significant interaction between group size and depletion treatment (LR‐χ^2^ = 0.0004, df = 1, *p* = 0.983).

**FIGURE 2:**
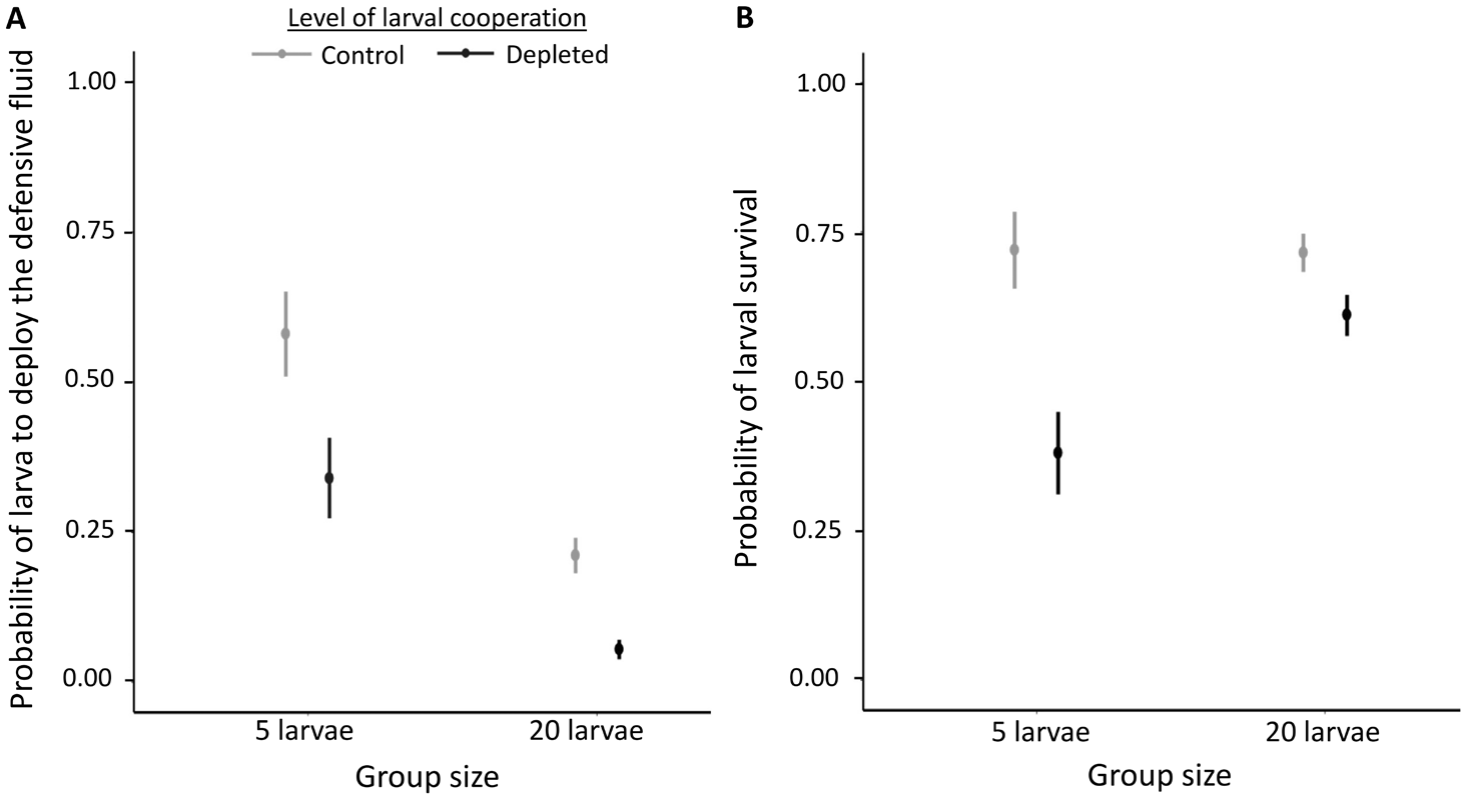
Effect of the group size (5 larvae or 20 larvae) and the level of cooperation (control or depleted) on collective defence and larval survival. (A) The probability of larvae deploying the defensive fluid is higher in small groups than in large groups (*N* = 10 for both group sizes). Depleted larvae are less likely to deploy the defensive fluid than control larvae (*N* = 10 for both treatments). (B) Depletion decreased the survival especially in the small groups (*N* = 10 for all treatments).

However, depletion significantly decreased larvae's likelihood to deploy the fluid in comparison to non‐depleted control larvae (*β* = −1.18, 95% ICr = [−1.69, −0.71], Figure 2A). Larvae in larger groups were also less likely to deploy fluid than those in smaller groups (*β* = −1.58, 95% ICr = [−2.04, −1.12], Figure 2A) with no significant interaction between group size (*β* = −0.93, 95% ICr = [−1.96, 0.09]). Thus, depletion treatment specifically reduced fluid deployment (i.e., public good) but did not limit participation for the U‐posture.

When assessing individual survival against ant predation in relation to group size and depletion treatment, we found a significant interaction between these two factors (LR‐χ^2^ = 14.02, df = 1, *p* = 0.0002, Figure 2B). This indicates that the presence of depleted (i.e., less defending) individuals had a stronger negative impact on survival in smaller groups than in larger ones (Figure 2B). In general, survival of individuals was lower in depleted groups in comparison to control groups (LR‐χ^2^ = 9.65, df = 1, *p* = 0.002), and in small groups in comparison to large groups (LR‐χ^2^ = 4.16, df = 1, *p* = 0.041).

### 3.2 Individuals Contribute Less to the Costly Collective Defence in Larger Than in Smaller Groups

**FIGURE 3:**
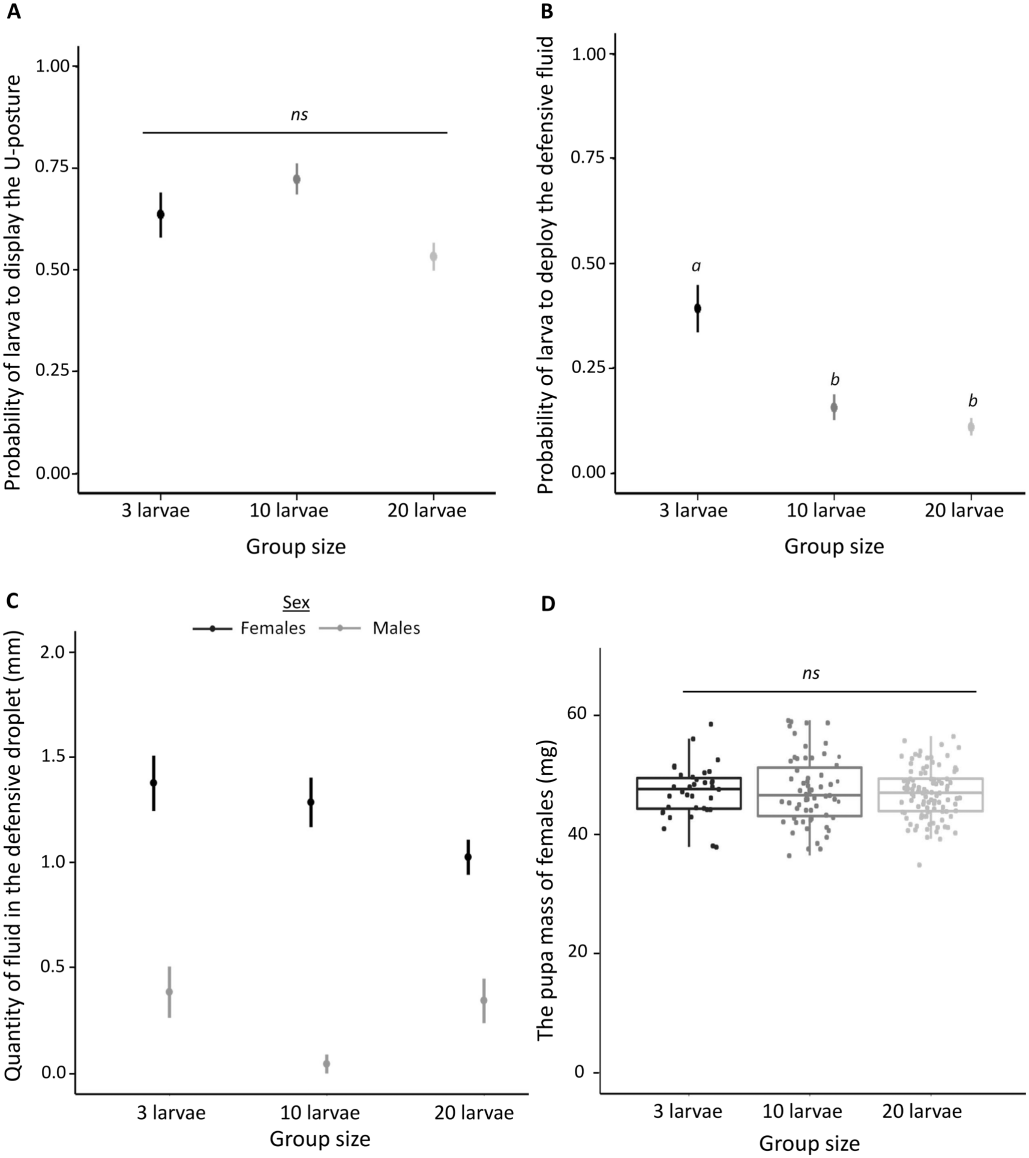
Effect of the group size on collective defence, individual defence and females' pupal mass. (A) The probability of exhibiting the defensive U‐posture after a simulated attack is similar in all group sizes. (B) The probability of deploying defensive fluid after a simulated attack is higher in groups of 3 larvae than in larger groups (10 or 20 larvae). (C) Females produce a higher volume of the defensive fluid than males. Especially, males from the group of 10 larvae produce a lower volume in comparison to other group sizes. (D) Females have a similar pupal mass even when reared in different sized groups. The box plots represent the median and interquartile range, with whiskers extending to 1.5 times the interquartile range; each point represents one individual. ns = no significant difference between treatments. For pairwise comparisons, different letters correspond to *p* < 0.05.

We first measured how the group size affected the defensive behaviour of the individuals within the group. There were no significant differences in the probability of larvae displaying the defensive U‐posture among group sizes (LR‐χ^2^ = 3.67, df = 2, *p* = 0.16, Figure 3A). When using the absolute number of larvae per group adopting U‐posture, we found that among larger groups more larvae displayed the U‐posture (LR‐χ^2^ = 94.55, df = 2, *p* < 0.0001); groups of 3 versus 10 larvae (*Z* = −7.41, *p* < 0.0001), groups of 3 versus 20 larvae (Z = −9.68, *p* < 0.001) and groups of 10 versus 20 larvae (Z = −2.67, *p* = 0.02) (Figure S1A).

The probability of deploying defensive fluid differed among group sizes (LR‐χ^2^ = 14.32, df = 2, *p* = 0.0008). More specifically, the larvae in larger groups (10 and 20) were less likely to contribute to collective defence than those in groups of 3 (groups of 3 vs. 10 larvae: Z = 2.91, *p* = 0.01; groups of 3 vs. 20 larvae: Z = 3.41, *p* = 0.002; groups of 10 vs. 20 larvae: Z = 0.36, *p* = 0.93, Figure 3B). However, the absolute number of larvae deploying the defensive fluid per group did not differ across group sizes (LR‐χ^2^ = 3.82, df = 2, *p* = 0.15, Figure S1B), suggesting that the total number of larvae committing to costly collective defence behaviour remained on the same level across group sizes.

Additionally, we assessed individual‐level contributions to chemical defence by attacking each larva separately (i.e., the larva defended itself). We found that the group size had a significant effect on the fluid deployment; individuals were less likely to deploy defensive fluid in groups of 10 larvae than in groups of 3 larvae (β = 1.51, 95% ICr [0.13, 3.37], Figure S2A). We found no clear evidence for a difference in fluid deployment between larvae in groups of 10 and in groups of 20 (*β* = −0.41, 95% ICr [−1.85, 0.82], Figure S2A). Sex also influenced deployment of the defensive fluid at the individual level; female larvae were more likely to deploy the defensive fluid than males (*β* = −1.25, 95% ICr = [−2.28, −0.28], Figure S2B).

When analysing the volume of fluid deployed, we found a significant interaction between the group size treatment and sex (*β* = −2.34, 95% ICr = [−4.34, −0.37], Figure 3C): in the group size treatment of 10 larvae, males deployed lower volumes of fluid in comparison to males from groups of 3 larvae and 20 larvae (*β* = 2.68, 95% ICr = [0.72, 4.46] and *β* = −2.63, 95% ICr = [−4.48, −0.93], respectively) or in comparison to volumes deployed by females under different group size treatments (Figure 3C). Again, females deployed higher volumes of fluid than males (*β* = −1.47, 95% ICr = [−2.86, −0.07]).

### 3.3 Life‐History Costs of Contributing to the Collective Defence Did Not Depend on the Group Size

The lower contribution to the collective defence in larger groups was not associated with differences in life‐history traits for an individual (Table 1): for both sexes, the developmental time, pupal mass or reproductive output did not depend on the group size treatment or individual's contribution to collective defence (i.e., whether the larva deployed the defensive fluid during individual defensive measurements or not) or the interaction between these two factors (Table 1: both sexes, Figure 3D, Figure S3: females only).

**TABLE 1:** Effect of the group size, defensive behaviour and their interaction on larval performance of males and females separately.

	Group size		Defensive behaviour		Group size × Defensive behaviour	
	LR χ^2^_2_	*p*	LR χ^2^_1_	*p*	LR χ^2^_2_	*p*
(a) Males' life history traits						
Larval developmental time	1.85	0.40	0.28	0.60	3.78	0.15
Pupal mass	0.85	0.65	0.42	0.52	2.74	0.25
(b) Females' life history traits						
Larval developmental time	4.15	0.13	0.04	0.84	0.04	0.98
Pupal mass	0.79	0.68	2.23	0.14	2.06	0.36
Number of eggs produced	2.59	0.27	1.52	0.22	0.0006	0.98

## 4 Discussion

Cooperation in public goods is expected to be more likely to evolve when group sizes are smaller due to increased net benefits and lower competition between group members (Lehmann and Keller 2006; Ross‐Gillespie et al. 2009). Here we show that cooperation in collective chemical defence provides greater survival benefits in smaller groups while freeloading incurs lower survival costs in larger groups. We also show that individuals modified their contributions to collective defence accordingly, by contributing less to costly chemical defence when in large groups. The lower contribution was not explained by constraints in resource availability, as larvae were fed *ad libitum* and no group size‐dependent effects were detected on life history traits such as developmental time, pupal mass or fecundity. Therefore, these results suggest that individuals can adjust their investment in costly collective defence according to its group‐size‐dependent benefits.

One possible explanation for the higher benefits of defending in smaller groups is that the higher *per capita* risk of attack increases the individual benefits of defending as individuals able to deploy the fluid are more likely to survive (Codella and Raffa 1996; Ritter et al. 2025; Skelhorn and Ruxton 2006). Alternatively, higher investment in costly chemical defence is likely to have a stronger survival effect in small groups, where each individual's contribution has a larger impact on defence effectiveness. These two explanations are not mutually exclusive and likely to function simultaneously (see also Ritter et al. 2025). Density‐dependence in contribution to collective chemical defence was also supported by the behavioural data (Figure 3A,B): the probability of individual participating in the more costly defence fluid deployment decreased in larger group sizes. This suggests that collective chemical defence in *N. sertifer* may continue to be effective as long as a critical number of individuals contribute in the vicinity of the attacked individual. In larger groups, this threshold can be reached with a smaller proportion of contributors, allowing more individuals to adopt a freeloading strategy where they can allocate resources for growth without compromising their survival (i.e., freeloading remains advantageous at the individual level even when its consequences for group defence may be less severe). This is also consistent with theoretical predictions that benefits from dilution effects at larger group sizes can be countered by reduced individual investment in costly defence, resulting in similar survival across group sizes (Figure 2, Daly et al. 2012). In public goods, such flexibility is expected to be favoured because it allows individuals to adjust their level of investment in response to environmental factors, such as group size, resource availability, or the other group members' behaviour (Daly et al. 2012; Solowiej‐Wedderburn et al. 2024).

However, the opposite trend was observed for the less costly U‐posture as the probability of larvae performing the defensive U‐posture was similar across group sizes (Figure 3A) and did not depend on the depletion status of an individual (see Section 3). This suggests that benefits of participating in the U‐posture may increase more linearly with increase in larval group size (Figure S1A). Participation in the U‐posture is also not constrained by the defensive status of the individual (e.g., whether it has fluid left or not, see Section 3). Thus, even though ‘posing’ in a defensive posture may trade off with the time available for feeding, the benefit‐to‐cost ratio of participating can be high and explain the lack of strong density‐dependence.

Cooperation in chemical defence in larger groups can also decrease due to the heightened associated life‐history costs; for example, due to increased competition for food (Daly et al. 2012). However, our data did not provide strong support for this. Larval development time, pupal mass and female egg production were unaffected by group size even when we considered individual's contribution to chemical defence in the last larval instar (i.e., if costs of being in a bigger group would only be visible in individuals that deployed and lost the fluid). This indicates that the life‐history costs of defence did not differ across treatments. These results could be explained by our experimental conditions (i.e., food *ad libitum*) that may have minimized the potential costs. However, our field observations suggest that, at least in the early stages of outbreak, there are only one‐to‐two larval groups within each pine tree, suggesting that food competition is low or non‐existent. During high density periods, food competition may however operate, and our experimental design therefore reflects conditions typical of low population density. Pine sawfly larvae also require help from others in feeding especially in smaller instars (Ghent 1960). The benefits of group‐facilitated feeding in larger groups could therefore have diminished the potential effects of resource competition.

In line with previous studies (see also Knerer 1984; Lindstedt et al. 2018; Ritter et al. 2025), we found that the contribution to collective defence is female‐biased both in terms of probability to deploy the fluid under attack as well as its volume. The female‐biased contribution was consistent across group sizes and not associated with higher life‐history costs for males (Table 1). One possible explanation is sexual size dimorphism, as females exhibit an additional larval instar and are larger than males (Giertych et al. 2007; Lindstedt et al. 2018; Wagner and Raffa 1993). Larger size could, for example, allow females to simply store and produce more defensive fluid, or experience lower relative costs when deploying it (Lindstedt et al. 2018). The asymmetry in costs of defending could then promote cooperativeness in the sex that pays lower costs (Gupta et al. 2025). Furthermore, pine sawfly populations are female‐biased (Coppel and Benjamin 1965; Craig and Mopper 1993; Ritter et al. 2025) and recent theoretical work focusing on sex‐biased peer‐to‐peer helping in the pine sawfly system suggests that when combined with female‐biased sex ratios, female helping can evolve even when the costs of helping are higher for females than for males (Gupta et al. 2025).

We do not have a clear explanation for why males reduced their contribution to defence in groups of 10 larvae. Other studies with pine sawflies suggest that individuals' contributions to collective defence decrease in male biased groups (Ritter et al. 2025). However, in our experiment the sex‐ratios did not differ between group size treatments (see Section 2) suggesting that sex‐ratio variation between treatments does not explain the observed pattern. Similarly, genetic variation was controlled across treatment groups using a split‐family design, and both food availability and abiotic conditions were standardised between treatment groups. Nevertheless, how (and why) female and male larvae change their behaviour under different social and ecological contexts will provide an interesting avenue for future studies.

Finally, group size effects on benefit:cost ratio of cooperation have been suggested to play a critical role in the transition from simple group living to more complex cooperation within a group (Groenewoud et al. 2016; Hamilton 1971). Accordingly, our results demonstrate that variation in group size shapes the benefit:cost ratio of cooperation among group members. This results in variation in the strength of selection toward cooperativeness and, in doing so, maintains diversity in its expression within and between groups. More generally, our results contribute to the growing body of empirical evidence suggesting that decreased predation risk promotes group living and its interaction with group size can be major ecological drivers of complex cooperation (Groenewoud et al. 2016; Krausz 2013).

## Author Contributions

C.L. conceived the ideas and C.L., R.R. and H.H. designed methodology; R.R. and C.L. collected the data; S.V.M. and R.R. analysed the data; S.V.M. led the writing of the manuscript, with input from R.R. and C.L. All authors contributed critically to the drafts and writing and gave final approval for publication.

## Funding

This work was supported by Research Council of Finland (257581 and 330578).

## Conflicts of Interest

The authors declare no conflicts of interest.

## Supporting information


**Appendix S1:** ele70378‐sup‐0001‐AppendixS1.zip.

## Data Availability

All data and final R code used in the analyses are archived in DRYAD https://doi.org/10.5061/dryad.jq2bvq8pc.
