# Supplementary material for: Group Size Dependent Selection for Cooperation Versus Freeloading in Collective Chemical Defence
Source: Ecol Lett. 2026 Apr 16;29(4):e70378. doi: 10.1111/ele.70378 (PMC13087209; doi:10.1111/ele.70378)
Supplement: Supplementary file 1 — Appendix S1: ele70378‐sup‐0001‐AppendixS1.zip. [file ELE-29-0-s001.zip › ele70378-sup-0001-FigureS1-S3-VideoS1-S2@13.03_Supplementary material.docx]

**Supplementary material**

**Group size dependent selection for cooperation versus freeloading in collective chemical defence**

Sophie Van Meyel, Raphael Ritter, Heikki Helanterä and Carita Lindstedt

**Supplemental material and methods**

*S1. N. sertifer laboratory population for group size effects on collective defence and life-history traits and collective defence*

To obtain the outbred F1 generation used in the experiment, we used individuals originating from 86 wild larval colonies collected from Central Finland (58 colonies, Puumala 62.066704, 27.351608 and 28 colonies, Pieksämäki 62.066704, 27.351608) and reared until adulthood at constant temperature (20 °C) and density (20 larvae per container) with fresh food (*Pinus sylvestris*) available *ad libitum*. Cocoons were individually stored in Petri dishes that were kept in the same rearing conditions as larvae. After adults eclosed, females were allowed to mate (one male per female) and lay their eggs on a randomly chosen pine branches on a living tree (*P. sylvestris*) (Central Finland 60º 15’ 17,48” N, 26º 1’ 59,03” E). Each branch with a mated pair was covered with a mesh bag. Females oviposit eggs inside pine needles and *N. sertifer* overwinters in the egg stage (Davis *et al.* 2023). Eggs die if the surrounding needle dies (Benjamin *et al.* 1955). Therefore, we had to use living trees and keep egg branches outdoors through the winter to allow the eggs to go through the obligatory diapause and develop.

*S.2 Statistical details*

*Effect of group size and levels of cooperation on larval survival against predators.*

The probability of displaying the U-posture (1 = yes, 0 = no) was analysed with a generalized linear mixed-effects model (GLMM) with a binomial error distribution and a complementary log-log (cloglog) link function. We used this link due to the moderate imbalance between 0 and 1 outcomes in the response variable. Due to singularity issues in the GLMM, we adopted a Bayesian approach using the brms package (Stan) as these models better accommodate low group-level variance and imbalanced data. Priors and Hamiltonian Monte Carlo settings are detailed below.

In each Bayesian model, we used weakly informative priors to regularize parameter estimation: normal (0,5) for fixed effects, student_t (3, 0, 10) for the intercept, and cauchy(0,5) for random effect standard deviations. The model was fitted using Hamiltonian Monte Carlo (HMC), with four chains of 4000 iterations.

All analyses were conducted in R v4.4.1 with the following packages: *car* (Fox & Weisberg 2011), *lme4* (Bates *et al.* 2015); *ggplot2* (Wickham 2016); *DHARMa* (Hartig 2016), *emmeans* (Lenth 2018)*, brms* (Bürkner 2017)*.* Model diagnostics were visually examined (Zuur et al. 2010), using the testResiduals() function from the DHARMa package, which performs standard diagnostic checks based on simulated residuals (Hartig 2016). In Bayesian models, convergence was evaluated by ensuring Rhat values were below 1.01 for all parameters and effective sample sizes (ESS) were sufficient, with no divergent transitions detected. Posterior predictive checks confirmed good model fit (Bürkner 2017).

**Supplemental figures**

**
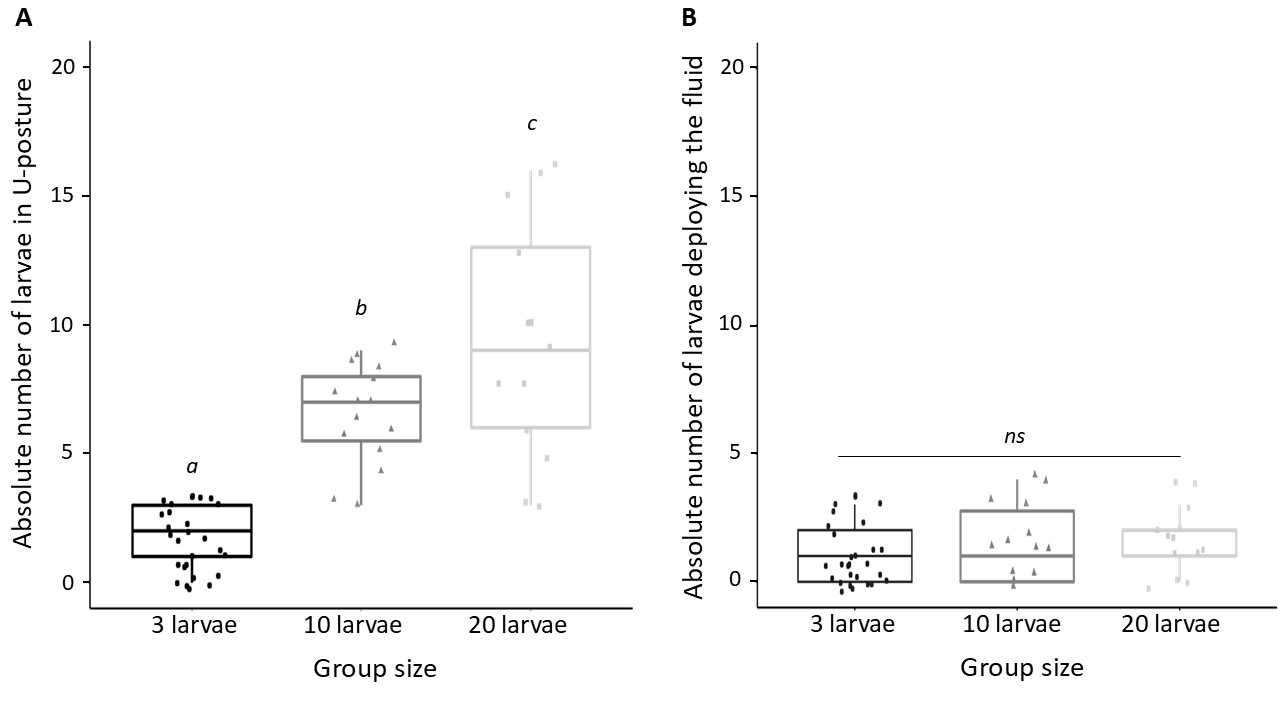
**

**Figure S1.** Effect of the group size on collective defence. (A) The absolute number of larvae exhibiting the defensive U-posture after a simulated attack is lower in groups of 3 larvae than in groups of 10 larvae and groups of 20 larvae. (B) The absolute number of larvae deploying the defensive fluid after a simulated attack is similar across all the group size. The box plots represent the median and interquartile range, with whiskers extending to 1.5 times the interquartile range, each point represents one individual. ns = no significant difference between treatments. For pairwise comparisons, different letters correspond to p < 0.05.

**
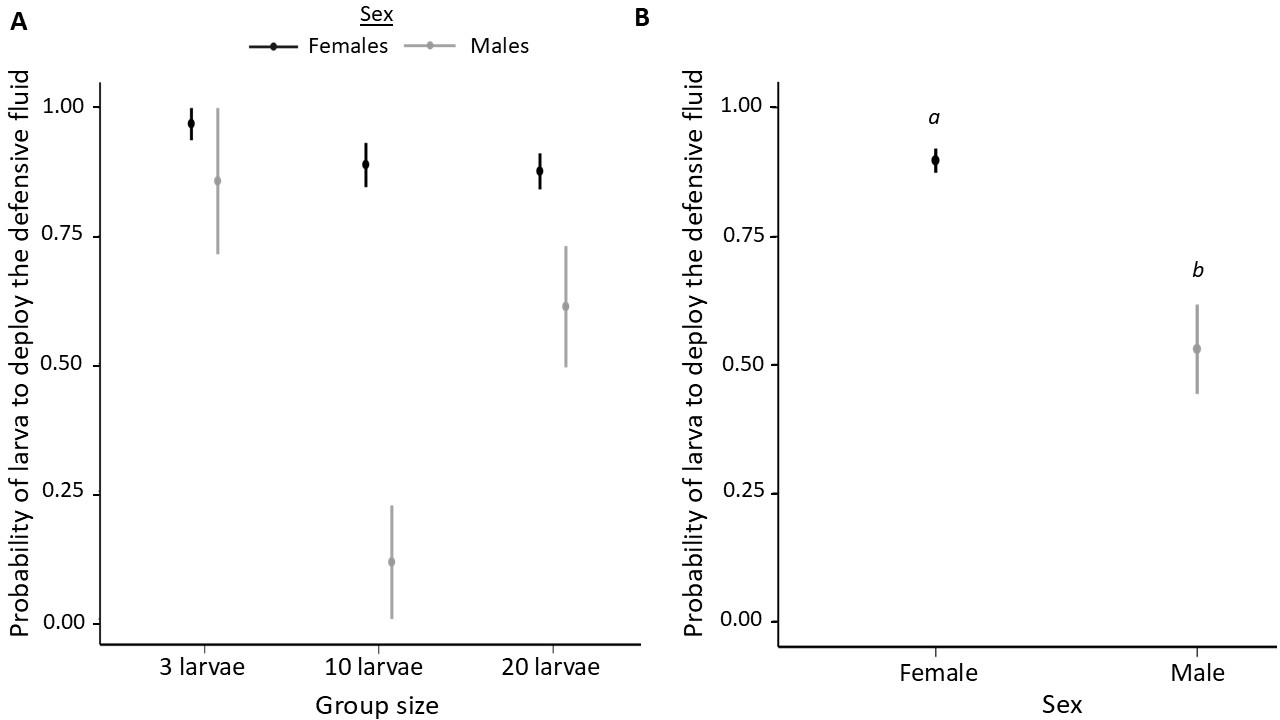
**

**Figure S2**. Effect of larval sex on individual defence behaviour. (A) Defensive fluid deployment was lower in groups of 10 larvae than in groups of 3 larvae but did not differ from groups of 20 larvae. (B) Females are more likely to deploy the defensive fluid than males. For pairwise comparisons, different letters indicate contrasts for which the 95% credible intervals do not overlap with zero.

**
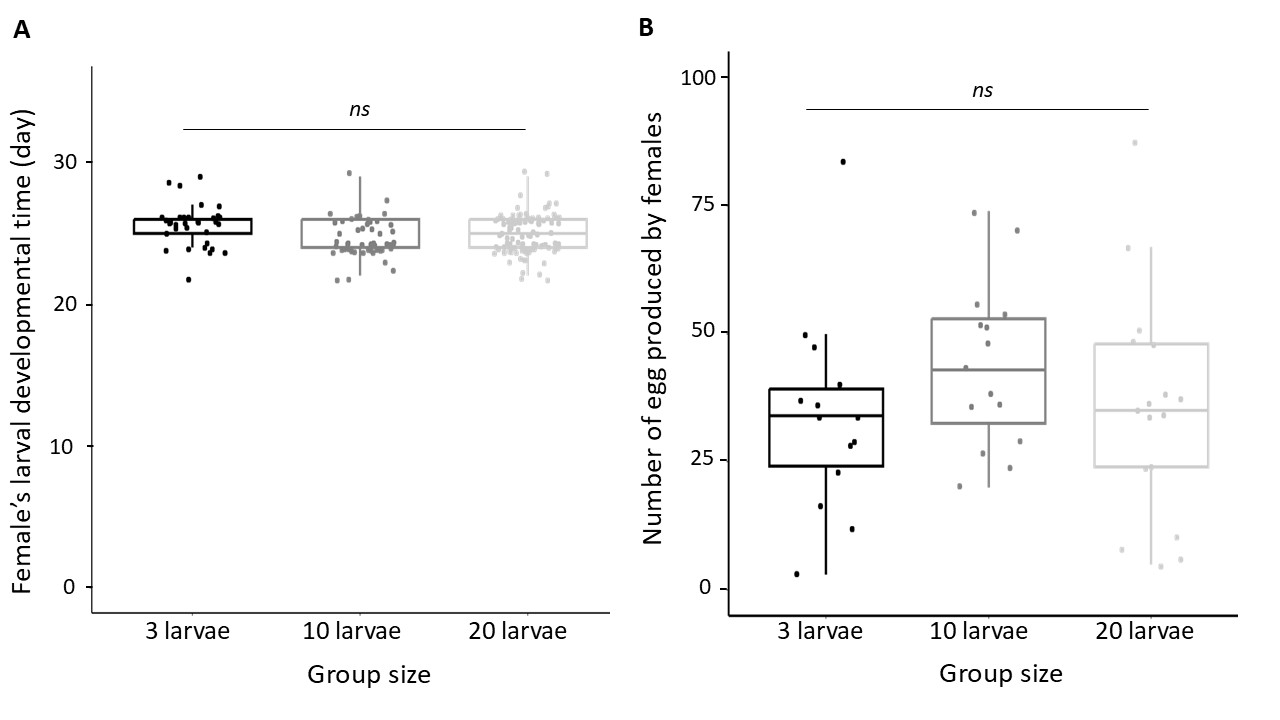
**

**Figure S3**. Effect of group size on females’ life-history traits. (A) Female larvae raised in groups of 3, 10 and 20 larvae develop equally fast, and (B) lay a similar number of eggs irrespective of the natal group size. The box plots represent the median and interquartile range, with whiskers extending to 1.5 times the interquartile range, each point represents one individual. ns = no significant difference between treatments.

**Supplementary Videos**

**Video S1**. Defensive display of mid-instar *Neodiprion sertifer* larvae showing the U-posture and fluid deployment.

**Video S2.** Defensive display of late-instar *Neodiprion sertifer* larvae (slow motion) illustrating the U-posture and fluid deployment.

**Supplemental references**

Bates, D., Mächler, M., Bolker, B. & Walker, S. (2015). Fitting Linear Mixed-Effects Models Using *lme4*. *Journal of Stastistical Software*, 67, 1-48.

Benjamin, D., Larson, J. & Drozz, A. (1955). The European pine sawfly on the Henderson state forest, Illinois, with notes on its biology and control. *J For*, 53, 359–362.

Bürkner, P.-C. (2017). *brms :* An R package for Bayesian multilevel models using *Stan*. *J Stat Softw*, 80, 1-28.

Davis, J.S., Glover, A.N., Everson, K.M., Coyle, D.R. & Linnen, C.R. (2023). Identification, biology, and management of conifer sawflies (Hymenoptera: Diprioninae) in eastern North America. *J Integr Pest Manag*, 14.

Fox, J. & Weisberg, S. (2011). *An R Companion to Applied Regression*. 2nd edn. Sage Publications, Thousand Oaks, CA.

Hartig, F. (2016). DHARMa: Residual Diagnostics for Hierarchical (Multi-Level / Mixed) Regression Models. *R package version 0.1.5.*

Lenth, R. (2018). emmeans: Estimated Marginal Means, aka Least-Squares Means. *R package version 1.1.2.*

Wickham, H. (2016). Programming with ggplot2. *In: ggplot2: elegant graphics for data analysis*, 2nd edn (ed. Wickham, H.). Springer, New York, NY, pp. 241–253.

Zuur, A.F., Ieno, E.N. & Elphick, C.S. (2010). A protocol for data exploration to avoid common statistical problems. *Methods Ecol Evol*, 1, 3–14.
